# Visualizing *Drosophila* centrioles by expansion microscopy

**DOI:** 10.1242/jcs.264338

**Published:** 2026-01-19

**Authors:** Emma E. Burns, Anastasia Amoiroglou, Carey J. Fagerstrom, John M. Ryniawec, LingSze Lee, Rose K. Runyan, Leah F. Rosin, Gregory C. Rogers, Nasser M. Rusan

**Affiliations:** ^1^ Cell and Developmental Biology Center, National Heart, Lung, and Blood Institute, National Institutes of Health, Bethesda, MD 20892, USA; ^2^ Department of Cellular and Molecular Medicine, University of Arizona Cancer Center, University of Arizona, Tucson, AZ 85721, USA; ^3^ Unit on Chromosome Dynamics, Division of Development Biology, Eunice Kennedy Shriver National Institute of Child Health and Human Development, National Institutes of Health, Bethesda, MD 20892, USA

**Keywords:** Centriole, *Drosophila*, Expansion microscopy, Procentriole

## Abstract

A significant challenge in studying the biology of the *Drosophila* centriole is its small size. Advanced super-resolution techniques have provided valuable insights but require specialized equipment and can be difficult to implement in tissues. Expansion microscopy (ExM) offers an accessible alternative, yet its application in *Drosophila* centriole research has been sparse. We provide an ExM protocol for cultured S2 cells and fly tissues that reveals new insights into procentriole biology. In S2 cells we document overduplication in the form of the classic ‘rosettes’, while in spermatids we uncover an unexpected movement of the procentriole-like structure (PCL). ExM has also refined existing molecular models. In S2 cells we document the distal tip protein Cep97 as a ring, which clarifies its role in capping the growing centriole. In spermatids, we spatially segregate the inner nuclear membrane protein Spag4 and the cytoplasmic protein Yuri, leading to the new hypothesis that they play independent roles at the centriole–nucleus contact site. Finally, we show that our ExM protocol is a hypothesis generator and discovery tool applicable beyond *Drosophila* centrioles by imaging synaptonemal complexes in the *Plodia interpunctella* moth.

## Teaser

**Summary:** We provide a detailed yet simple expansion microscopy protocol for *Drosophila* cultured cells and tissues. We also report new insights into various aspects of centriole biology.

## INTRODUCTION

Centrosomes are organelles that function as major microtubule-organizing centers that are used to build microtubule-based molecular machines such as cilia and mitotic spindles (Aljiboury and Hehnly, 2023; Fernandes-Mariano et al., 2025). Centrosomes are composed of barrel-shaped centrioles surrounded by a scaffold of pericentriolar material (PCM), which serves as a docking site for γ-tubulin ring complexes that nucleate microtubule growth (Vasquez-Limeta and Loncarek, 2021). Most cells contain a single centrosome that, like DNA, duplicates once during S phase to ensure that cells enter mitosis with two centrosomes (Ryniawec and Rogers, 2021). Due to their role in organizing the mitotic spindle, centrosomes are critical for high-fidelity chromosome segregation (Fernandes-Mariano et al., 2025; Zhao et al., 2021). Centrioles, the core structural components of centrosomes, perform a second critical role as basal bodies that form and anchor primary and motile cilia at the plasma membrane (Breslow and Holland, 2019; Kobayashi and Dynlacht, 2011). Importantly, variants of centrosome and cilia genes are associated with microcephaly, lissencephaly, polydactyly and primordial dwarfism, as well as a host of ciliopathies (Bettencourt-Dias et al., 2011; Elliott and Brugmann, 2019). Thus, cells exert tight control over centrosome numbers, structure and function to prevent pathological initiation and progression.

The fruit fly (*Drosophila melanogaster*) is a powerful model system for studying centrosome biology and has contributed greatly to our understanding of centrosome function in development and dysfunction in disease. Centrioles in most fly cells are approximately half the length of human centrioles, but they are similar in diameter and structure (Greenan et al., 2018). Both human and fly centrioles contain a unique set of evolutionarily conserved proteins that regulate duplication and contribute to their structure and function (Fernandes-Mariano et al., 2025). Importantly, the majority of the key proteins that comprise the *Drosophila* centriole have clear human homologs (Carvalho-Santos et al., 2011). One major obstacle to studying *Drosophila* centrosomes is their small size (∼200 nm in length in most cell types). Advances in super-resolution microscopy (SRM) over the past decade have greatly enhanced our understanding of protein localization within centrosomes as well as their mechanisms of recruitment (Fu and Glover, 2012; Jana et al., 2018; Lawo et al., 2012; Mennella et al., 2012; Sonnen et al., 2012; Tian et al., 2021; Vlijm et al., 2018). However, the most common and accessible SRM techniques such as structured illumination microscopy (SIM) and stimulated emission depletion microscopy (STED) provide 50–100 nm lateral (*x-y*) resolution and 150–500 nm axial (*z*) resolution under ideal conditions, which is insufficient to accurately resolve fly centriole ultrastructure (Jana et al., 2018). Moreover, these techniques often rely on imaging overexpressed GFP-tagged proteins, which could force the localization to non-physiological sites. To overcome these obstacles, the centrosome field continues to adopt expansion microscopy (ExM; Chang et al., 2017; Chen et al., 2015) and its variations such as ultrastructure expansion microscopy (U-ExM; Gambarotto et al., 2019), ExM combined with SRM methods (Wang et al., 2024) and iterative ultrastructure expansion microscopy (iU-ExM; Louvel et al., 2023), as cost-effective and accessible techniques to investigate protein localization at a resolution of 60 nm or less. The benefits and applications of ExM have been reviewed previously (Gallagher and Zhao, 2021; Gambarotto et al., 2021; Hümpfer et al., 2024; Tillberg and Chen, 2019; Wassie et al., 2019; Zhuang and Shi, 2023).

ExM has been used to investigate *Drosophila* embryos (Jiang et al., 2018; Parveen et al., 2023), both larval (Jiang et al., 2018; Steib et al., 2022) and adult tissues (Bullard et al., 2024; Cahoon et al., 2017; Dubey et al., 2022; Gao et al., 2019; Lin et al., 2022; Mosca et al., 2017; Wainman, 2021; Wang et al., 2018), and cultured *Drosophila* cells (Tian et al., 2021). While the use of ExM has been rapidly increasing for studying centrosomes (Ching et al., 2022; Le Guennec et al., 2020; Sahabandu et al., 2019; Scott et al., 2023), its use to study *Drosophila* centrioles has been limited to only a few studies to date (Brunet et al., 2025; Steib et al., 2022; Wainman, 2021).

Here, we adopt centriole-specific ExM protocols for use in *Drosophila* cultured cells and tissue (Gambarotto et al., 2021; Kong and Loncarek, 2021; Sahabandu et al., 2019). We show that this method enables the clear visualization of inner centriolar proteins, reveals novel procentriole dynamics in both cells and tissues, and allows us to refine the molecular architecture of the centriole and its associated proteins.

## RESULTS

### Expansion microscopy can improve antibody epitope recognition

**Fig. 1.**
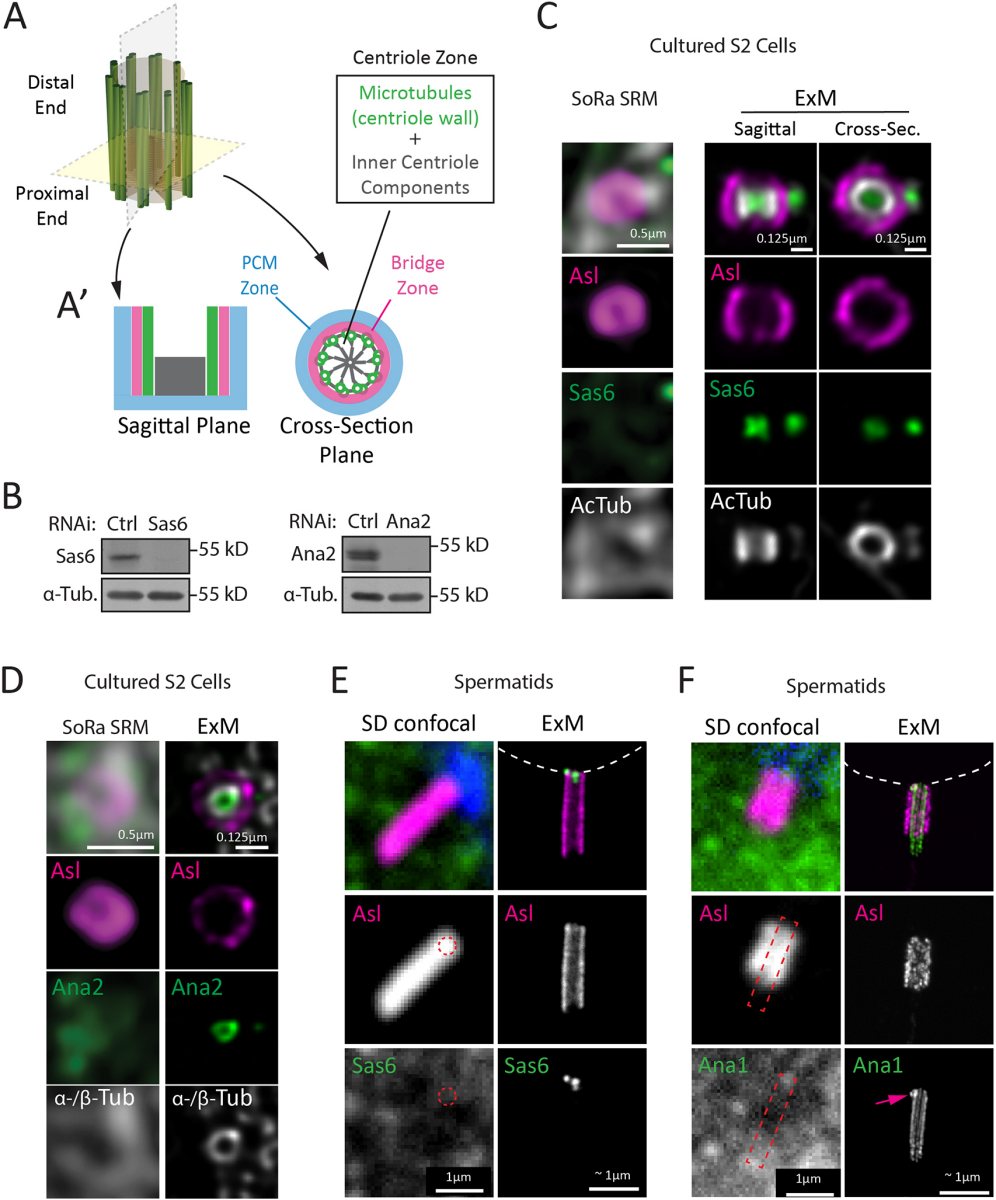
**ExM greatly improves epitope recognition.** (A) Cartoon of a centriole showing nine sets of two or three microtubules (green). Centriole microtubule polarity conveys a proximal–distal identity. The cartwheel is a proximal structure (red). (A′) The centrosome is radially organized into three zones: (1) the centriole zone, containing microtubules (green) and internal structures (gray); (2) the bridge zone (magenta); and (3) the PCM zone (blue). Shown are sagittal and cross-sectional views. (B) Western blots of Sas6 and Ana2 in S2 cells depleted with control (Ctrl), Sas6 or Ana2 RNAi for 7 days. α-Tubulin (α-Tub), loading control. Blots shown are representative of three experiments. kD, kilodalton. (C) SoRa SRM of S2 cell centrosomes stained for Asterless (Asl, magenta), Sas6 (green) and acetylated tubulin (AcTub; gray) prepared using cold methanol fixation (left column), or ExM (middle and right columns). Sagittal and cross-sectional views of centrioles are shown for ExM. (D) SoRa SRM of S2 cell centrosomes stained for Asl (magenta), Ana2 (green) and α/β-tubulin (α-/β-Tub; gray) prepared using cold methanol fixation (left column), or ExM (right columns). Images shown in C and D are representative of >10 experiments and >100 centrioles. Scale bars in C and D: SoRA SRM, 0.5 μm; ExM, 0.125 μm. S2 cell expansion factor was calculated as 4× using the ratio of pre- and post-expanded gels. (E,F) Spinning-disk (SD) confocal microscopy of spermatid GC stained for Asl (magenta) and either Sas6 (E; green) or Ana1 (F; green) using conventional fixation (left columns) and tissue ExM (right columns). Nuclei are shown in SD images by DAPI (blue). Nuclei in ExM images indicated by white dashed outline. The PCL (pink arrow) is located at the proximal end of the GC. Red dashed circle in E indicates where Sas6 is predicted to be based on Asl staining. Red dashed rectangle in F indicates where the centriole is predicated to be based on Asl staining. Scale bars in E and F: 1 μm for SD confocal and ∼1 μm for ExM. Spermatid expansion factor was calculated as 5.2× using known diameter of Asl and Ana1 (see Materials and Methods). Images in E and F are representative of five experiments.

Centrosomes can be subdivided into three concentric functional zones: (1) the centriole zone, which contains proteins that localize within the centriole core; (2) the PCM zone, which contains scaffolding proteins that surround the centriole barrel; and (3) the bridge zone, which contains proteins that tether PCM proteins to the centriole surface and often extend between the microtubule bundles (Fig. 1A; Varadarajan and Rusan, 2018). One major obstacle in *Drosophila* centrosome biology is the scarcity of antibodies that can recognize the centriole proper (centriolar microtubules), in addition to the difficulty in identifying many of the inner centriole components (Fig. 1A). For example, the centriole is templated by an internal component termed the cartwheel, which is formed by stacks of rings composed of nine Sas6 dimers with outward-facing coiled-coil domains that form the spokes of the cartwheel (Gopalakrishnan et al., 2010; Nakazawa et al., 2007). In human cells, the cartwheel is a transient structure, as Sas6 is normally removed from new daughter centrioles via ubiquitin-mediated proteolysis during mitotic exit (Strnad et al., 2007). In contrast, the Sas6 cartwheel is a permanent structure within fly centrioles (Callaini et al., 1997). Additional inner centriole proteins, such as Cep135, localize along the carboxy-terminal region of Sas6, which corresponds to the distal tips of the spokes, appearing as an electron-dense structure called the pinhead (Fig. 1A) (Lin et al., 2013). These precede the assembly of microtubules to establish the ninefold radial symmetry of the centriolar microtubule bundles (Hiraki et al., 2007; Lin et al., 2013). Our labs have historically struggled to generate antibodies that detect proteins in the cartwheel and the pinhead by immunofluorescence (IF). However, using ExM has drastically changed the outcome. We posit that expanding the centriole might expose epitopes that could then be detected by our antibodies.

We previously raised antibodies against the N-terminus of Sas6 (Rogers et al., 2008), which forms the central hub of the cartwheel (Kitagawa et al., 2011; van Breugel et al., 2011). Western blots using this antibody revealed a single distinct band in S2 cell lysates that is depleted after 7 days of Sas6 RNA interference (RNAi) (Fig. 1B). Despite its specificity, this antibody shows a diffuse signal that does not colocalize with Asl-labeled rings using conventional IF with a cold methanol fixative (Fig. 1C, SoRa SRM). Similarly, immunostaining for acetylated tubulin was unreliable; colocalization with Asl was observed in several cases but the staining was irregular (Fig. 1C, SoRa SRM). However, using ExM, we clearly detected centrioles using an anti-acetylated tubulin antibody, and consistently observed a strong Sas6 signal both at the center of the mother centriole and within the procentriole (Fig. 1C, ExM). Notably, the Sas6 cartwheel stacks vertically through the centriole (Guichard et al., 2017), occupying most of the centriole lumen (Fig. 1C, ExM). Similar to S2 cells, successful Sas6 antibody detection was also observed in whole-tissue ExM of *Drosophila* testes. In comparison with conventional spinning-disk confocal microscopy, where no Sas6 was detected (Fig. 1E, SD confocal), ExM revealed two distinct Sas6 signals. One Sas6 spot corresponds to the cartwheel of the *Drosophila* ‘giant centriole’ (GC; the term is used for *Drosophila* spermatid centrioles) and the second signal corresponds to the procentriole-like (PCL) structure, an atypical procentriole that serves as a template for the first daughter centriole formation following egg fertilization (Fig. 1E, ExM; Jo et al., 2019).

Similar success was achieved with antibodies against other inner centriole components. Antibodies against Ana2 (the ortholog of human STIL) behaved similarly to our anti-Sas6 antibodies; they demonstrated specificity by western blot but not for IF using conventional methods (Fig. 1B,D). However, with ExM, rings of Ana2 were observed within the centriole lumen and juxtaposed to the inner centriole wall as identified by antibodies to α/β-tubulin, which also showed exceptional improvement over conventional IF (Fig. 1D) (Tian et al., 2021). We also saw similarly significant improvement in spermatids using an antibody against Ana1 (the ortholog of human Cep295). Ana1 is positioned across the centriole microtubules and is recognized as a linker between the inner centriole components and bridge proteins (Fu and Glover, 2012; Galletta et al., 2016; Tian et al., 2021; Tsuchiya et al., 2016). With ExM, our antibody against Ana1 clearly recognized the GC as a barrel structure (Fig. 1F, ExM) with the newly formed PCL at its proximal end (Fig. 1F, arrow).

Although we do not understand why these antibodies are successful for IF following sample expansion, we speculate that the protocol either removes steric barriers to proteins in dense regions of the centriole or exposes epitopes as a result of denaturation. While this highlights an advantage of ExM, it is important to note the possibility of ExM disrupting antigen epitopes, resulting in failure of antibody recognition. Our labs have not faced this limitation, but it is well documented elsewhere (Gambarotto et al., 2021; Ku et al., 2016). Regardless, using ExM has greatly increased the questions we can tackle surrounding a wider range of centriole proteins.

### Expansion microscopy reveals new insights into *Drosophila* procentriole biology

**Fig. 2.**
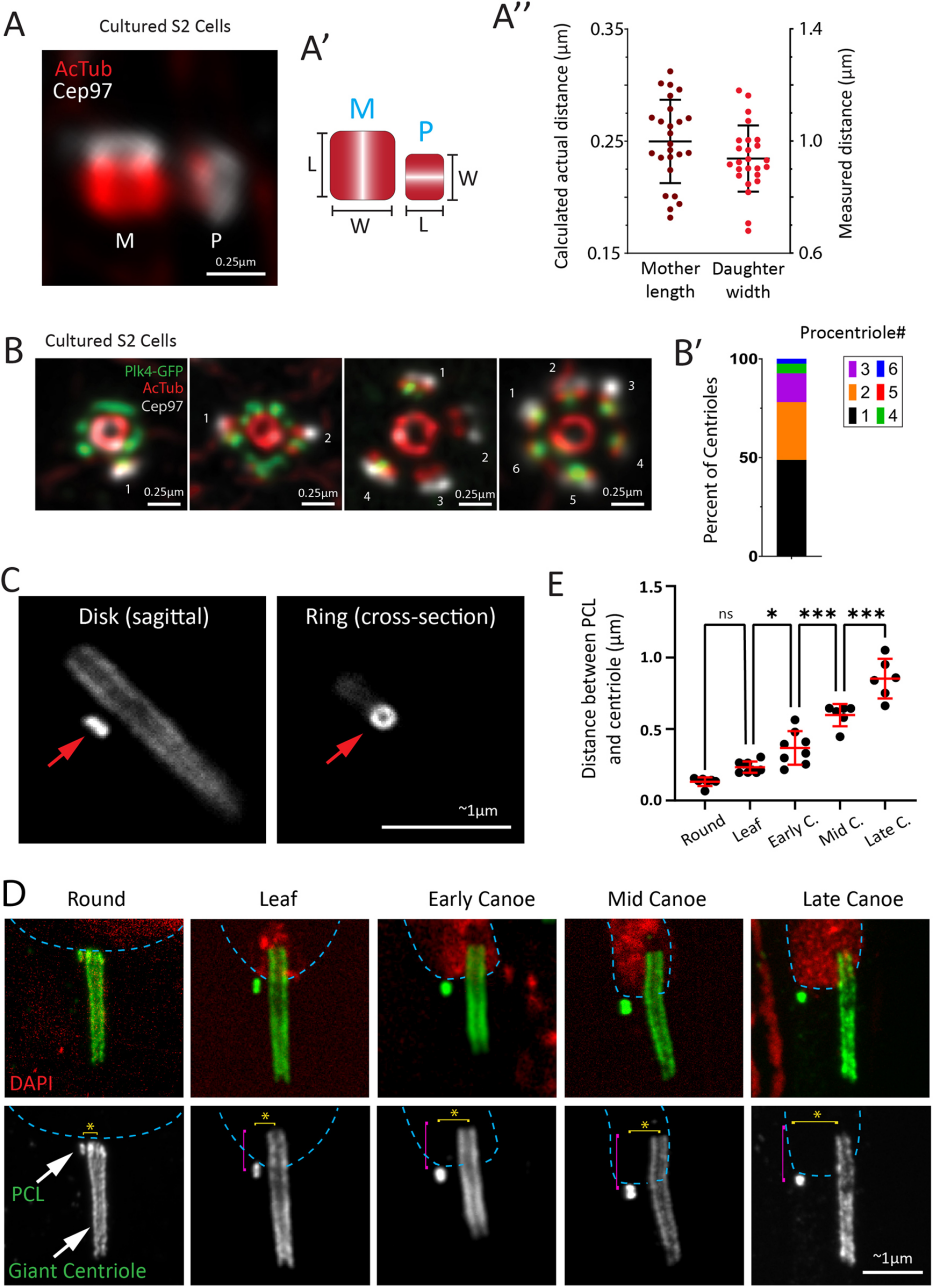
***Drosophila* procentriole biology can readily be investigated using ExM.** (A) ExM of S2 cells immunostained for acetylated tubulin (AcTub; red) and the centriole distal tip protein Cep97 (gray). M, mother centriole; P, procentriole. (A′) Cartoon depicting measurement of the length (L) of the mother centriole and the width (W) of the procentriole. (A″) Distance measurements of mother centriole length and procentriole (daughter) width. Right *y*-axis indicates the measured distance, while the left *y*-axis indicates the calculated distance based on the expansion factor of 4×, thus indicating a true biological measurement. *n*=24 total centrioles from three independent experiments. (B) ExM of S2 cells overexpressing Plk4–GFP and immunostained for GFP (green), acetylated tubulin (red) and Cep97 (gray). The image gallery shows a mother centriole with various numbers of attached procentrioles. (B′) Graph showing the number of procentrioles per mother. *n*=41 centrosomes from three independent experiments. Scale bars in A and B: 0.25 μm. (C) ExM of wild-type spermatids expressing Ana1::GFP. Shown is both a sagittal view of the PCL as a disk (red arrow, left) and a cross-sectional view of the PCL as a ring (red arrow, right). (D) ExM images showing the spermatid GC and PCL (immunostained for Ana1, green) at the indicated developmental stages. The PCL is initially at the proximal end of the GC near the nucleus (DAPI, red) and is progressively positioned more distally (pink bracket), and perpendicularly further away (yellow bracket and asterisk) from the GC as spermatids develop. During these movements, the PCL always remains adjacent to the nucleus (blue dashed line). (E) Quantification of the distance between the PCL and centriole at round elongating (*n*=7), leaf (*n*=9), early canoe (Early C.; *n*=8), mid canoe (Mid C.; *n*=6); and late canoe (Late C.; *n*=6) stages, as shown in D. Statistical comparisons were made using a one-way ANOVA with Tukey's correction (ns, not significant; *P<0.0332; ***P<0.0002). Scale bars in C and D: 1 μm for SD confocal and ∼1 μm for ExM. Spermatid expansion factor was calculated as 5.2× using known diameter of Asl (see Materials and Methods). Error bars represent the mean±s.d.

Given the small size of *Drosophila* centrioles (∼ 200 nm×200 nm) and the difficulty of detecting microtubules within the centriole wall, it has been challenging to accurately measure the dimensions of mother and daughter centrioles, as is typically done in mammalian cells. However, with improved resolution and antibody recognition using ExM, we were able to consistently detect and measure both the length of mother centrioles and width of their procentrioles. We used antibodies against acetylated tubulin to detect the centriole wall and against Cep97 to confirm the distal centriole tip position (Fig. 2A,A′; Dobbelaere et al., 2020). Our measurements indicated that the mother centriole averaged 249 nm in length when corrected for expansion factor, whereas the procentriole width was roughly 235 nm (Fig. 2A″). The length of the procentriole was not reported, as it varied considerably, likely due to capturing the procentriole at various stages of elongation, as has been previously shown in S2 and Hela cells (Kong et al., 2020; Raynaud-Messina et al., 2004). Another obstacle faced by *Drosophila* centriole researchers due to the short length of the centrioles is the inability to detect centriole duplication errors. In normal cells, centrosome number is maintained through a semi-conservative centriole duplication cycle that is coupled to the cell cycle (Nigg and Holland, 2018). Overexpression of Plk4, the master regulator of centriole duplication, induces supernumerary centrioles as a single mother centriole templates multiple daughters simultaneously, a common occurrence in cancer cells (Bettencourt-Dias et al., 2005; Habedanck et al., 2005; Hamzah et al., 2025; Kiermaier et al., 2024). In human cells, the phenomenon of centriole amplification can be detected by conventional IF and standard confocal microscopy, which reveals a ‘rosette’ configuration of a mother centriole surrounded by several engaged procentrioles (Habedanck et al., 2005; Kleylein-Sohn et al., 2007). Overexpression of Plk4 (also known as Sak in *Drosophila*) in S2 cells also induces centriole amplification by stimulating the assembly of multiple procentrioles on individual mother centrioles. However, due to the shorter length of fly procentrioles, rosettes have not been seen in S2 cells using classic IF techniques. Rather, centriole amplification is detected after the multiple daughters have separated (disengaged) from their mothers. Recently, ExM has been used to reveal rosettes in *Drosophila* male germline cells expressing non-degradable Plk4 (Brunet et al., 2025). In an attempt to detect rosettes in S2 cells, we overexpressed Plk4–GFP and followed our standard ExM protocol. We stained cells for acetylated tubulin and Cep97 after expansion, which revealed a classic rosette structure of mother centriole with multiple attached daughters (Fig. 2B). The maximum number of procentrioles that we detected on the mother centriole was six (Fig. 2B,B′). This maximum differs from that of seven in human cells (Sullenberger et al., 2023), but the biological significance, if any, of this difference is unknown at this point.

Similar to cultured S2 cells, studies of procentrioles in *Drosophila* tissues have been quite difficult due to the same size and resolution limitations mentioned above. One particular difficulty we have faced is investigating the behavior of the PCL structure in spermatids (Blachon et al., 2009). The PCL is challenging to study because it lacks the microtubule wall found in a typical centriole. However, it does contain canonical centriole components such as Sas6, Ana2 and Ana1, which appear to form a cylinder (with no internal cartwheel) surrounded by electron-dense material (Khire et al., 2016). Thus, the PCL is akin to a juvenile centriole in a state of arrested development, only to be used in the zygote following *Drosophila* fertilization (Blachon et al., 2014). More recently, the PCL has been shown to be critical in properly anchoring the GC to the nucleus at the latest stages of spermiogenesis (Buglak et al., 2024). Interestingly, the PCL has been shown to be highly dynamic, moving from the extreme basal end of the GC toward the center of the GC, a distance of ∼800 nm (Buglak et al., 2024). This PCL translocation is coupled to a highly dynamic event where the GC is indented into the nucleus to form the ‘centriole cap’, uncovering the first known instance of a procentriole traveling along a mother centriole.

We were curious to see whether ExM could uncover any additional details of PCL movement. We dissected, fixed and expanded adult male testes expressing Ana1::GFP, which appeared as a disk from the sagittal view and a ring from a cross-sectional view (Fig. 2C). Thus, the PCL is indeed a cylindrical structure as previously described (Khire et al., 2016). We then imaged spermatids at five developmental stages during which the GC is known to invaginate into the nucleus. The movement of the PCL toward the distal end of the mother centriole was clearly seen (Fig. 2D, pink bracket) (Buglak et al., 2024). Surprisingly, the increased resolution of ExM revealed a second PCL movement, in this case, perpendicular to the GC (Fig. 2D, yellow bracket, E). This movement suggests that the PCL is not engaged with the GC but rather engaged with the nuclear envelope. A similar perpendicular movement called ‘distancing’ has been documented in cultured mammalian cells using electron microscopy (Shukla et al., 2015) and recently using ExM (Takeda et al., 2024). This second movement of the PCL is a great demonstration of how ExM is an important, accessible imaging technique that breaks the conventional resolution barrier and leads to new hypotheses.

### Expansion microscopy reveals critical protein localization details

**Fig. 3.**
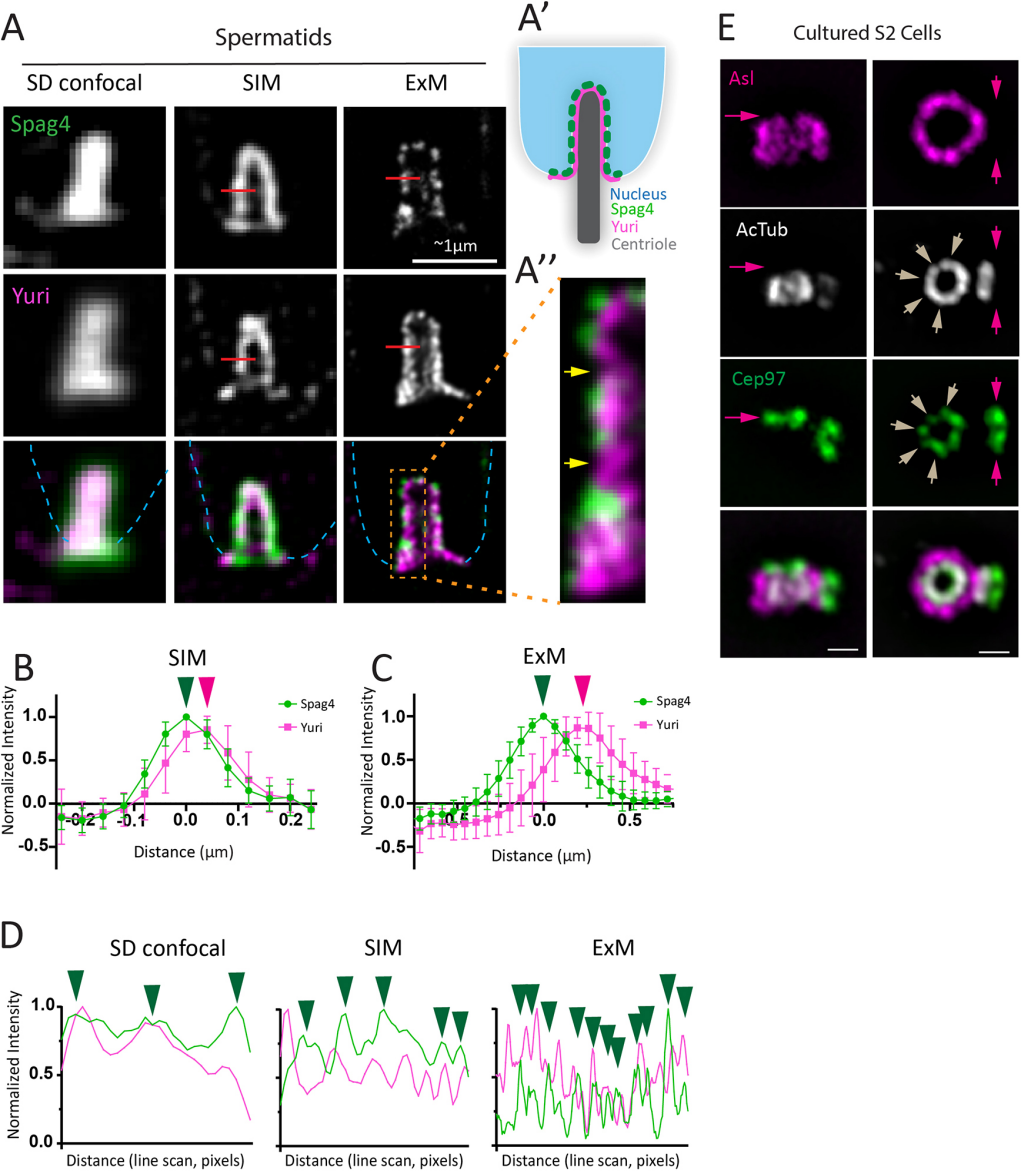
**ExM microscopy reveals unappreciated details of *Drosophila* centriole biology.** (A) Spermatids showing the site of centriole invagination into the nucleus. Comparison between spinning disk (SD) confocal, SIM and ExM. Tissue stained for Spag4 (green) and Yuri (magenta). Nuclei are indicated by the blue dashed outlines. Red lines show positions of line scans as in B. Dashed box marks the region shown in A″. (A′) Cartoon representation of Spag4 and Yuri forming the centriole cap that encases the proximal end. (A″) ExM reveals the punctate nature of Spag4 that is positioned more internally to the nucleus (not stained). Yuri is closer to the centriole and does not fully overlap with Spag4, suggesting an additional linker that might reside in the indicated gaps (yellow arrows). Scale bars: 1 μm for SD confocal and SIM and ∼1 μm for ExM (calculated based on the known width of the Asl from previous studies and thus a close approximation of true biological distance). (B) Line scan averages across the nuclear membranes from SIM images (A, red line) indicate the inability to distinguish Spag4 and Yuri localization. *X*=0 was determined by the highest Spag4 peak. *n*=13 for Spag4, *n*=13 for Yuri. (C) Line scan averages from ExM images (A, red line) indicate that Spag4 and Yuri are spatially related but distinct. *X*=0 was determined by the highest Spag4 peak. *n*=11 for Spag4, *n*=11 for Yuri. Arrowheads in B and C indicate peak values for Spag4 (green) and Yuri (magenta). Error bars represent the mean±s.d. (D) Representative line scans along the nuclear membrane at the centriole cap region in SD, SIM and ExM imaging as in A. The increase in the number of peaks indicates the higher spatial detail of Spag4 (green arrowheads) and Yuri localization due to the increased resolution of ExM. (E) Cultured S2 cells imaged using ExM and immunostained for Asl (magenta), acetylated tubulin (AcTub; gray) and Cep97 (green). Cep97 localization is seen as a ring at the distal tip of both the mother centriole and procentriole (pink arrows). ExM reveals the repeated punctate pattern of Cep97 that likely matches the ninefold symmetry of the centriolar microtubules (gray arrows). Left, sagittal view; right, cross-section view. Images are representative of >10 experiments and >40 centrioles. Scale bars: 0.125 μm. S2 cell expansion factor was calculated as 4× using ratio of pre- and post-expanded gels.

Following our successful new insight into the behavior of the PCL, we wondered whether ExM could further help investigate the biology of GC invagination into the spermatid nucleus. Our previous study has shown that the inner nuclear membrane (INM) Sun-domain protein Spag4 and the cytoplasmic protein Yuri form the ‘centriole cap’ around the proximal end of the GC (Fig. 3A) (Buglak et al., 2024). With conventional confocal microscopy and with SIM, it appeared that Spag4 and Yuri were in the same spatial position (Fig. 3A,B), even though they are predicted to be in different cellular compartments. However, using ExM, we were able to spatially separate Spag4 and Yuri, showing that Spag4 is indeed in the INM and does not colocalize with Yuri (Fig. 3A,C). However, these new images challenge our previous hypothesis that Spag4, via an unknown intermediate, anchors Yuri at the centriole cap. We found that Spag4 localization is sparse and irregular, which does not match the more densely localized Yuri (Fig. 3A ExM, A′ compare green dashes and solid magenta, A″ yellow arrows, D). Thus, a linear molecular linkage of Spag4–Kash protein–Yuri model seems insufficient. We now hypothesize that an unknown component of the nuclear membrane serves as the key anchor for Yuri on the cytoplasmic side and Spag4 on the INM. Current experiments are underway to address this hypothesis.

ExM has the potential to revise our centriole models in S2 cells; one example being our studies of the centriole distal end. Centrioles have inherent polarity along their length that is established during procentriole assembly, with the proximal end facing the mother centriole and the distal end pointing away from the mother. Centrioles grow processivity from their distal tip, where a conserved set of proteins, known as the distal tip complex (or DTC), regulates growth of the centriole microtubules (Ryniawec et al., 2023). Cep97 is the foundational component of the DTC and is quickly recruited to the nascent procentriole during the G1-S phase transition (Ryniawec et al., 2023; Spektor et al., 2007). Indeed, we observed that all procentrioles in S2 cells show a Cep97 cap at the distal end (Fig. 3E). Using 3D-SIM and STED, we have previously found that Cep97 localizes to centriole distal tips, appearing as a single spot at the center of a centriole in cross-section (Dobbelaere et al., 2020; Ryniawec et al., 2023; Tian et al., 2021). However, at that level of resolution, and because of the use of markers outside of the centriole microtubule barrel (i.e. Asl and PLP), it is unclear with which centriolar substructures Cep97 might associate. Using ExM, we found that Cep97 decorates the distal tip as a ring of punctate spots that overlap solely with acetylated microtubules, although the exact number of spots was unclear (Fig. 3E). Thus, sample expansion reveals that *Drosophila* Cep97 is not a uniform cap that covers the entire centriole tip but rather localizes on top of the microtubule doublets that comprise the centriole wall. These results are consistent with the proposed role of Cep97 in limiting centriole microtubule growth and its localization in ExM of human cells (Dobbelaere et al., 2020; Laporte et al., 2024).

## DISCUSSION

ExM has transformed our ability to resolve biological structures at previously inaccessible nanoscale resolutions, presenting a groundbreaking shift in the landscape of microscopy-based research. Building upon foundational ExM studies in *Drosophila*, our simplified ExM protocols for cultured S2 cells and fly tissues significantly enhance the detection of centriolar proteins such as Sas6, Ana1 and Ana2.

Our work not only demonstrates improved imaging clarity but also uncovers novel biological phenomena. For example, we can clearly observe centriole overduplication and ‘rosette’ formations in S2 cells, highlighting the potential for ExM to detect nuanced aberrations in centriole duplication. Furthermore, in spermatids, our application of ExM reveals previously unappreciated lateral movements of the PCL structure, suggesting novel interactions with the nuclear envelope that ensure proper sperm head–tail connection. These findings illustrate the transformative potential of ExM in uncovering subtle, yet biologically significant dynamics.

Our work and simplified protocol also showcases how ExM can quickly be added to any biologist's toolkit, allowing them to refine their molecular models, as we have shown with for centriole duplication and dynamics. To demonstrate the ease of following this protocol, we sought to test ExM on non-centrosomal biology and in two additional systems – the *Drosophila* ovary and the moth *Plodia interpunctella* testis. In both systems, conventional confocal microscopy cannot resolve the pairs of axis-associated proteins, such as transverse filament proteins or lateral element proteins that reside on either side of a central element within meiotic synaptonemal complexes (see schematics in Fig. S1). These transverse filaments and lateral elements form along the chromosome axis for both homologous chromosomes and thus reside all along the meiotic bivalents as pairs. The distance between the C termini of the transverse filament protein C(3)G is 100 nm, and thus not resolved in traditional whole mounts, but is clearly seen using ExM (Fig. S1A; Cahoon et al., 2017). Similarly, the distance between the parallel lateral elements of paired meiotic chromosomes in *Plodia* testes is only resolved by ExM (Fig. S1B). The implementation of our protocol in these two new systems was accomplished the very first time it was attempted by the Rosin lab, highlighting the ease of protocol integration into a new lab space and encouraging further experimentation to molecularly map synaptonemal complexes in a range of species.

The use of ExM represents not just an enhancement in imaging capabilities but a true acceleration of discovery and hypothesis generation. As we continue to push the boundaries of magnification and molecular interrogation, we move closer to achieving a better understanding of cellular machinery, revolutionizing our insights into cellular function and dysfunction.

## MATERIALS AND METHODS

### Expansion microscopy – *Drosophila* S2 cell protocol

The U-ExM protocol was adapted from Kong and Loncarek (2021) for S2 cell expansion. Note that for simplicity throughout this protocol we use the term ExM even though the original protocol describes centriole-magnified analysis of the proteome (c-MAP; Sahabandu et al., 2019).

#### Materials

- 25 mm round coverslips
- 6-well tissue culture plate or single 35 mm round plastic dishes
- Humidity chamber comprising a closed container with wet paper towels inside
- 10 cm plastic Petri dishes
- 4 mm biopsy punch tool (Integra; 33–34-SH)
- 50 ml conical tubes
- Hot plate
- 1 l glass beaker
- Flour sifter with 0.6 mm mesh hole
- Sodium acrylate
  o Note: the quality of sodium acrylate varies between suppliers and lots. Using poor-quality sodium acrylate can cause the formation of spherical occlusions in polymerized gels (Kong et al., 2024).
- 24-deep-well plate (Bio Trend; 931568)

#### Stock reagents

- Sf900 II serum-free medium (Thermo Fisher Scientific; 10902088) supplemented with 1× penicillin streptomycin solution (Corning; 30-002-CI)
- 0.5 mg/ml concanavalin A (ConA) in H_2_O
- 10× phosphate-buffered saline (PBS)
- 32% paraformaldehyde (PFA) solution, EM grade (Electron Microscopy Sciences; 15714-S)
- Ice-cold histology-grade methanol
- PBS with 0.1% Triton X-100
- 40% acrylamide in H_2_O (20 g acrylamide, bring to 50 ml with H_2_O and store at 4°C)
- 40% acrylamide:bis-acrylamide (19:1) in H_2_O (1.9 g acrylamide and 0.1 g bis-acrylamide, bring to 5 ml with H_2_O and store at 4°C)
- 5% ammonium persulfate (APS) in 2× PBS (50 mg of APS, bring to 1 ml with 2× PBS, store at 4°C for a maximum of 2 weeks)
- Sodium acrylate (Pfaultz & Bauer; S03880)
- TEMED 99%, extra pure (Thermo Fisher Scientific; 138450500)
- Normal goat serum (NGS) blocking buffer (5% NGS in PBS with 0.1% TritonX-100)
- Bovine serum albumin (BSA) blocking buffer (1% BSA in PBS with 0.05% Tween 20)
- Hoechst 33342 (Thermo Fisher Scientific; H3570)
- 1 mM propyl gallate (0.212 g propyl gallate in 1 l H_2_O; enough for 20 expansions)
- 0.01% w/v poly-L-lysine (100 mg poly-L-lysine, bring to 1 ml with H_2_O and store at 4°C)
- VALAP sealant (1:1:1; melt together at low temperature 16.6 g of Vaseline, 16.6 g lanolin and 16.6 g paraffin wax for a final volume of ∼50 ml)

#### Expansion solutions

Freshly prepare the following expansion solutions before use.
• 4% PFA in 1× PBS
  o 1 ml 32% PFA stock and 7 ml 1× PBS; make fresh
• 17.72% sodium acrylate in 2× PBS
  o 280 mg of sodium acrylate in 1.44 ml 2× PBS; make fresh and store on ice
• Polymerization solution: 20% acrylamide, 0.04% bis-acrylamide, 7% sodium acrylate, 0.5% TEMED, 0.5% APS in 1× PBS
  o To make 4 ml Polymerization solution, add in a pre-cooled 15 ml conical tube: 1.920 ml 40% acrylamide, 0.08 ml 40% acrylamide:bis-acrylamide, 1.580 ml 20% sodium acrylate, 0.020 ml TEMED, 0.400 ml 5% APS in 2× PBS.
• Anchoring solution: 30% acrylamide, 4% PFA, 1× PBS
  o To make 8 ml Anchoring solution (2 ml per coverslip), add 6 ml 40% acrylamide, 1.5 ml 32% PFA, 1.5 ml 1× PBS; make fresh.
• Denaturation buffer: 50 mM Tris-HCl pH 9.0, 200 mM NaCl, 200 mM SDS
  o Prepare 1 l and store at room temperature.
• Expansion buffer: 1 mM propyl gallate in H_2_O
  o Prepare 1 l and store at 4°C; for 4× expansion.

#### Procedure for S2 cells

##### Day 1: plate and fix cells

1. Place one 25 mm round coverslip in a well of a 6-well plate or into one 35 mm dish. Coat coverslips with ConA by spreading 500 μl ConA solution around entire surface of the coverslips, then removing the entire volume. Allow coverslips to dry.
2. Resuspend confluent S2 cells by pipetting semi-adherent cells in growth medium (15 ml medium in T75 flask or 1.5 ml medium in 6-well plate).
3. Plate resuspended S2 cells densely on ConA-treated 25 mm round coverslips (75 μl of 100% confluent S2 cells in 100 μl fresh medium). Allow cells to spread until they flatten and become phase dark (between 45 min and 3 h).
4. Fix in 4% PFA (2 ml per coverslip) for 4 min at room temperature.
5. Remove PFA solution and post-fix in ice-cold methanol (2 ml per coverslip) for 4 min at −20°C.
  o Note: methanol enhances cell permeability and antigenicity for anti-centriole antibodies. This step might not be necessary in all cases and can be considered an optional variation of the protocol.
6. Rinse coverslips 3× with 1× PBS to remove all methanol and ensure no interference with the Anchoring solution.
7. Incubate coverslips in 1× PBS for 20 min at room temperature.
  o Note: cells can be stored in PBS at 4°C in a humidity chamber for months. Generally, we fix ∼6 coverslips per condition and process them for expansion immediately before imaging.
8. Incubate coverslips in Anchoring solution (2 ml per coverslip) overnight at 40°C in a humidity chamber.

##### Day 2: gel embedding, sample denaturation and cell staining

9. Rinse wells/coverslips 3× in 1× PBS to remove excess Anchoring solution.
10. Wash 2× in 1× PBS for 10 min at room temperature to equilibrate samples.
11. Prepare a 10 cm dish with a Parafilm insert and place in ice-water bath (Fig. 5B). Dry the back of coverslips with a Kimwipe and place the coverslips on Parafilm. Add 0.5 ml 1× PBS for 20 min to cool samples.
12. Prepare Polymerization solution (except APS) in a pre-cooled Eppendorf tube, vortex to mix thoroughly, and keep on ice.
  o Note: the acrylamide will begin to polymerize when you add APS, so do not add this yet. Additionally, ensuring slides and reagents are ice cold will slow polymerization rates, which is essential for consistent polymerization across sample.
13. Aspirate PBS from coverslips (remove as much liquid as possible).
14. Complete the Polymerization solution by adding APS. Quickly vortex to mix, and place on ice.
15. Add 1 ml Polymerization solution to each coverslip and gently pipet to mix without introducing bubbles, to ensure any residual PBS is mixed with the Polymerization solution.
16. Remove 0.6 ml Polymerization solution to create a thin and even layer and allow it to polymerize on ice for 20 min (Fig. 5B).
  o Note for steps 14–16: gels polymerize quickly after adding APS, so work efficiently.
17. Move the 10 cm dish from ice to room temperature for 30 min to complete polymerization.
18. Use the punch tool (Fig. 5A1) to excise four gel punches per coverslip (Fig. 5C,C′). Use forceps (Fig. 5A2) to place punches in a 35 mm dish (Fig. 5E).
19. Add 2 ml Denaturation buffer to each 35 mm dish and incubate for 15 min at room temperature.
20. Transfer gel punches to a 50 ml conical tube and add 25 ml Denaturation buffer (use one conical tube per coverslip). Incubate conical tubes in a boiling water bath for >1.5 h (Fig. 5F).
  o Note: denaturation is critical. Incomplete denaturation will lead to inconsistent immunostaining and can prevent some antibodies from working.
  o Note: this volume of Denaturation buffer is sufficient for 4–6 gel punches. We have not attempted this with only one punch, but if scaling down is necessary, then exercise caution when handling.
21. Dump conical tubes onto the flour sifter (one at a time, as to not mix samples) to remove Denaturation buffer and capture gel punches (Fig. 5H). Pour 1× PBS onto punches to rinse excess SDS.
22. Add 40 ml 1× PBS in a new 50 ml conical tube and add the gel punches. Rock the 50 ml conical tubes for 15 min at room temperature.
23. Repeat step 22 nine times to wash SDS from the gel punches.
  o Note: when washing with PBS, also rinse the gel with 1× PBS to ensure SDS is removed. If SDS is not fully removed, gel punches will freeze when placed at 4°C, and staining will not work.
24. Transfer punches, using a spatula (Fig. 5A3), to a 24-deep-well plate containing 400 μl NGS blocking buffer (Fig. 5I,I′). Shake for 30 min at room temperature.
  o Note: if using primary antibodies raised in goat, use BSA blocking buffer for blocking and primary antibody solution.
25. Stain punches in 400 μl primary antibody solution diluted in blocking buffer – place the 24-deep-well plate in a humidity chamber and shake on an orbital shaker at room temperature overnight.

##### Day 3: complete cell staining and gel expansion

26. Remove and retain primary antibody solution for subsequent staining.
27. Wash wells 3×10 min with 1 ml PBS containing 0.1% Triton X-100 (shaking).
28. Label punches in 400 μl secondary antibody and 3.2 Hoechst – place 24-deep-well plate in humidity chamber (cover in tinfoil for light protection) and shake at RT for 5 h (or overnight).
  o Note: if using primary antibodies raised in goat, use BSA blocking buffer for secondary antibody solution.
29. Wash 3×10 min with 1 ml PBS containing 0.1% Triton X-100 (shaking).
30. Transfer each gel punch to an aluminum foil-coated 50 ml conical tube (protected from light). Expand gel discs by incubating overnight at 4°C in 45 ml Expansion buffer.
  o Note: gel punches can be stored at 4°C in Expansion buffer (protected from light) until ready to image. To ensure the best imaging quality, expanded gel punches should be imaged within a week of expansion. In our experience, image quality deteriorates quickly, and we prefer to image on the next day.

##### Day 4: mounting sample and imaging

31. Treat a dish-mounted coverslip (i.e. MatTek dish) with poly-L-lysine by spreading 300 μl across the coverslip surface, letting sit for 5 min, removing the entire volume and allowing to dry.
32. Place each gel punch on a grid ruler and measure the diameter to calculate the expansion factor (Fig. 5K).
  o Note: determine which side of the expanded gel punch contains the cells by placing the gel on an untreated coverslip. Examine using a phase-contrast or fluorescence microscope with a 100× objective. Flip gel or change objective if necessary.
  o Note: if the gel is too thick to manipulate, you can use a clean razor blade to cut the expanded gel punch in half and use only the side with the embedded cells.
33. Dry the expanded gel punch as well as possible without touching the cells. Place the punch on a dry metal spatula so that cells are facing the spatula and use a Kimwipe to dry the top and sides of the gel. Angle the spatula to wick away excess liquid from the bottom of the gel.
34. Place the expanded gel with cells facing toward the coated coverslip. Trim excess gel so that the punch sits flat on the center of the coverslip without touching the plastic of the dish.
35. Gently press on the expanded gel to adhere to the glass-bottom dish and prevent the gel from sliding.
36. If you experience gel drift, add 1–1.5 ml melted VALAP around the gel to secure it on the dish while applying pressure with your finger. VALAP should be touching the glass 360° around the punch and creating a continuous coating around the top. Sealing will prevent the gel from drifting during imaging.
  o Note: melt VALAP at ∼60–65°C and use when fully melted. If VALAP is too hot, it will warm up the gel and cause it to lose water.
37. Gel punches are now ready to image. We tend to mount one punch at a time to prevent gel drying and signal deterioration.

### Expansion microscopy – *Drosophila* tissue protocol

The U-ExM protocol for *Drosophila* tissue was adapted from previously published protocols (Gambarotto et al., 2021; Wainman, 2021). Note that for simplicity throughout this protocol we use the term ExM.

#### Materials

- 1.5 ml tubes
- 12-well glass plate
- Forceps
- Humidity chamber comprising a closed dish with parafilm over wet filter paper
- 12 mm coverslips
- Spatula
- 35 mm round dishes
- Biopsy punch tool (4 mm diameter)
- Heat block
- 250 ml glass jars
- 24-well plate
- 250 ml amber glass jars
- Poly-L-lysine-coated glass-bottom dishes

#### Stock reagents

- 40% acrylamide (purchased at this concentration)
- 37% formaldehyde
- 10× PBS (80 g NaCl, 2 g KCl, 2 g KH_2_PO_4_, 21.1 g Na_2_HPO_4_•7H_2_O with 700 ml H_2_O, adjust pH to 7.3 and bring volume up to 1 l)
- 1× PBS (100 ml 10× PBS with 900 ml H_2_O)
- 2% *N*,*N′*-methylenebisacrylamide (purchased at this concentration)
- 0.5 M Tris pH 9.0 (60.55 g Tris base in 350 ml H_2_O, adjust pH to 9.0, then bring volume to 500 ml with H_2_O)
- 5 M NaCl (146.1 g NaCl in 500 ml H_2_O)
- 10% SDS
- 10% APS (0.1 g APS in 1 ml H_2_O; store as 100 μl aliquots at −20°C for up to a month)
- 10% TEMED (100 µl TEMED, 900 µl H_2_O; store as 100 μl aliquots at −20°C for up to a month)
- 38% sodium acrylate solution (19 g sodium acrylate, 31 ml H_2_O; filter and store at 4°C)
  o Note: sodium acrylate solution can be used until it turns yellow. Do not use if the solution is yellow!
  o Note: if the sodium acrylate precipitates from solution when preparing, then add more water dropwise until it resolubilizes.

#### Expansion solutions

Freshly prepare the following expansion solutions before use.
• Monomer solution (MS): 500 µl 38% sodium acrylate solution, 250 µl 40% acrylamide, 100 µl 10× PBS, 50 µl 2% *N*,*N′*-methylenebisacrylamide
  o Make 45 µl aliquots and store at −20°C (up to 2–3 weeks). Needs to be stored at −20°C at least overnight before use (see step 6, below).
• Denaturation buffer: 10 ml 0.5 M Tris pH 9.0, 4 ml 5 M NaCl and 57.14 ml 10% SDS
  o Stir and then bring to 100 ml with H_2_O. Store at room temperature.
  o Note: the quality of this reagent determines the success of the expansion. When in doubt make this reagent again.
• Anchoring solution (or FA/AA): 38 µl 37% formaldehyde, 50 µl 40% acrylamide and 912 µl 1× PBS.
  o This produces a 1.4% formaldehyde:2% acrylamide mixture.

#### Procedure for *Drosophila* tissue

##### Day 1: dissect and fix tissue

**Fig. 4.**
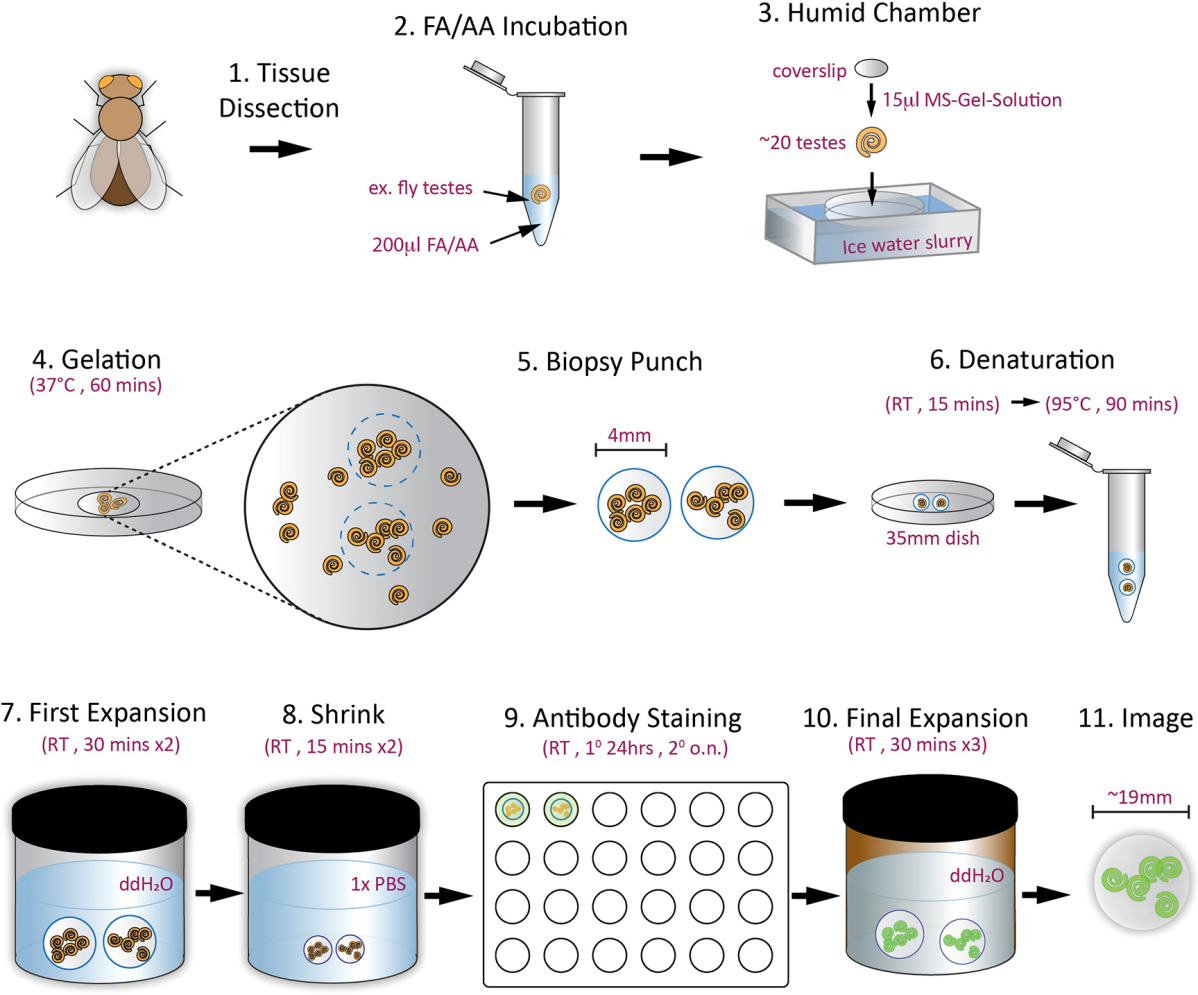
**Graphical illustration of ExM method for *Drosophila* tissue.** Step 1: the process begins with the dissection of testes (or other tissue), with ∼40 testes of the same genotype placed in each tube. Step 2: the tissue is then fixed overnight in an Anchoring solution (FA/AA). Step 3: samples (20 testes each) are embedded in a monomer solution on a pre-chilled humid chamber and then covered with a coverslip to initiate gelation. Step 4: polymerization is completed by incubating at 37°C. Step 5: a biopsy tool is used to punch out gel pieces that contain the embedded tissue. Step 6: gel punches are transferred to a dish and then a tube for protein denaturation (RT, room temperature). Step 7: the gels undergo the first expansion in water (ddH_2_O, double-distilled water). Step 8: gels are washed in PBS to shrink and prepare for staining. Step 9: gels are immunostained with primary (1°) and secondary (2°) antibodies (o.n., overnight). Step 10: a second expansion is performed by incubating the gels in water. Step 11: the fully expanded gel is mounted onto a glass-bottom dish for microscopy.

1. Dissect testes and transfer into a small tube containing Sf900 II (dissection medium). Place 40 testes per tube (Fig. 4 step 1). Use separate tubes for different genotypes.
2. Remove dissection medium and add 200 µl Anchoring solution (FA/AA) to each tube (Fig. 4 step 2). Incubate at 37°C overnight.

##### Day 2: gel polymerization, protein denaturation, first gel expansion and tissue staining

**Fig. 5.**
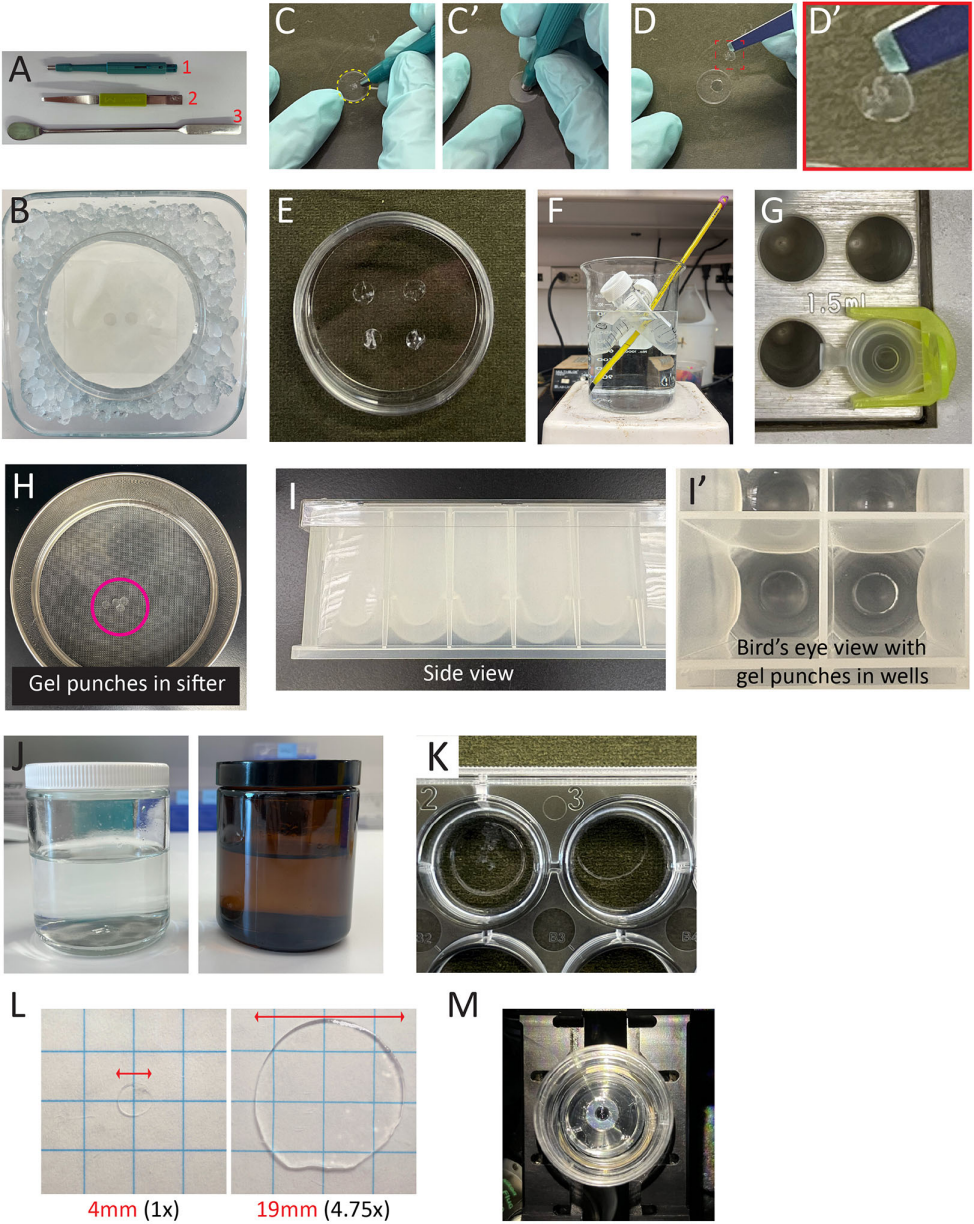
**Images of materials and methods for both cell and tissue protocols.** (A) Key tools used include a biopsy punch (1), forceps (2) and a spatula (3). (B) The gel polymerization setup, showing a coverslip on a Parafilm-lined dish in an ice-water bath to keep the reagents cold. (C,C′) Gels are excised from the coverslip using the biopsy punch tool. Dashed circle, gel. (D,D′) The resulting unexpanded gel punches are transferred with forceps. Dashed box, area enlarged in D′. (E) Punches are placed in a dish with Denaturation buffer. (F) For the S2 cell protocol, denaturation occurs by boiling punches in a conical tube heated in a water bath on a hotplate. (G) For the tissue protocol, denaturation is performed in 1.5 ml Eppendorf tubes in a heat block at 95°C. (H) For the S2 cells, gels are rinsed using a flour sifter. (I,I′) Gels are transferred to 24-deep-well plates for staining. Then gels are transferred to a 50 ml conical tube wrapped in aluminum foil (not shown) to perform the expansion step. (J) For the tissue protocol, gels are transferred to a 250 ml clear jar to perform the first expansion, then shrinking. An amber jar is used for the final gel expansion after immunostaining. (K) For the tissue protocol, shrunk gels are placed in a 24-well plate for staining. (L) Pre-expanded (left) and post-expanded (right) gels are placed on graph paper to measure the expansion factor. (M) Expanded gel is mounted in a glass-bottom dish and covered in water for imaging.

3. Prepare a humid chamber by placing filter paper in 150 mm plastic Petri dish to cover the bottom surface. Moisten the filter paper with distilled water. Place a 2×2 section of parafilm on the damp filter paper and put both the humid chamber and a metal tube holder (a heat block) at −20°C for 15 min.
4. Place the prepared humid chamber and a metal block on two separate ice water slurries (Fig. 4 step 3; Fig. 5B).
5. Retrieve tubes containing testes (or any tissue) out of the 37°C incubator. Transfer tissue and Anchoring solution (to avoid tissue drying out) from the tubes to a 12-well dish. Each well will contain tissue (∼20 testes of one genotype) prepared for one expansion gel.
6. Transfer MS, 10% TEMED and 10% APS from −20°C into a metal block holder on ice water slurry to keep cold.
  o Note: complete the next steps as quickly as possible to prevent gelation before the solution reaches the cells. Keeping the humid chamber and reagents cold will slow polymerization rates, which is essential for successful gel embedding across the sample.
7. Transfer testes with Anchoring solution from one well of the 12-well dish onto parafilm in a humid chamber as a single drop.
  o Note: it is best to transfer only two samples at a time, proceed to steps 8–10 for those two samples and then repeat with remaining samples from the 12-well dish until completed.
8. Aspirate Anchoring solution from the drop containing tissue (remove as much liquid as possible).
9. Make MS–Gel solution: add 2.5 µl 10% TEMED, followed by 2.5 µl 10% APS to a 45 µl MS aliquot, quickly vortex. (This deviates from referenced protocols, see note below.)
  o Note: the acrylamide will begin to polymerize when APS is added, so be sure to add TEMED to the MS aliquot before adding APS.
10. Immediately add a 15 µl drop of MS–Gel solution to the tissue in a humid chamber (remember to only work with two samples at a time). Then rapidly, but gently, place a 12 mm round coverslip on top of the drop (Fig. 4 step 3; Fig. 5B).
  o Note: this step deviates from the referenced protocol. The decreased volume of MS–Gel solution achieves a thinner gel, which keeps tissue within the microscope imaging plane.
11. Incubate on ice water bath for 5 min.
12. Transfer the humid chamber from ice to 37°C for 1 h to complete polymerization (Fig. 4 step 4).
13. Carefully flip the round coverslips. Use the biopsy punch tool (Fig. 5A1) to excise two gel punches per coverslip (Fig. 5C,C′).
  o Note: this step deviates from the reference protocol. Biopsy punching creates a gel with a smaller surface area that is easier to handle.
  o Note: tissue is visible at this point. Try to punch around tissue clusters to maximize samples (Fig. 4 step 5; Fig. 5C).
14. Use forceps (Fig. 5A2) to transfer punches (Fig. 5D,D′) of the same genotype into a 35 mm dish (Fig. 4 step 6; Fig. 5E).
15. Add 1 ml Denaturation buffer to each 35 mm dish and incubate at room temperature for 15 min with agitation (Fig. 4 step 6).
16. Use a spatula (Fig. 5A3) to transfer gel punches from each 35 mm dish to a 1.5 ml tube filled with 1.4 ml Denaturation buffer (Fig. 4 step 6).
17. Incubate in a heat block set at 95°C for 1.5 h (Fig. 5G).
  o Note: as stated in the S2 cell procedure, denaturation is critical and incomplete denaturation will lead to inconsistent immunostaining.
18. Pour contents of each tube into separate 250 ml lidded glass jars filled with ∼100 ml H_2_O, incubate at room temperature for 30 min (Fig. 4 step 7; Fig. 5J).
19. Exchange most of the H_2_O and incubate at room temperature for another 30 min.
  o Note: be careful not to lose the gels as they are hard to see at this point but should be getting bigger. Pour out the H_2_O into a 150 mm dish and check for gel pieces that escaped before discarding the water.
20. Exchange the H_2_O with 1× PBS (∼100 ml). Perform 2×15 min washes in PBS at room temperature (Fig. 4 step 8).
21. Transfer gels to a 24-well plate, place one gel per well (Fig. 4 step 9; Fig. 5K).
  o Note: to transfer gels, pour most of the PBS into a 150 mm dish, check for gel punches that escaped before discarding. Pour the remaining PBS containing the gel punches into the same 150 mm dish and use a spatula to place each into individual wells of the 24-well plate.
22. Add 250 µl of solution containing primary antibodies diluted in PBS containing Tween 20 (0.1%) and 5% NGS. Incubate for 24 h at room temperature with gentle agitation (Fig. 4 step 9) (guinea pig anti-Asterless, 1:5000; chicken anti-Ana1, 1:1000; chicken anti-Yuri, 1:100; rabbit anti-GFP, 1:500; rabbit anti-RFP, 1:250; Table S1).

##### Day 3: tissue staining (continued)

23. Remove primary antibody solution from gels.
24. Wash gels 3×10 min with 500 µl PBS containing Tween 20 (0.1%) at room temperature with gentle agitation.
25. Add 250 µl of solution containing secondary antibodies diluted in PBS containing Tween 20 (0.1%) and 5% NGS. Incubate overnight at room temperature with gentle agitation and protected from light (Fig. 4 step 9) (1:500 dilution).

##### Day 4: final gel expansion, mounting sample and imaging

26. Remove secondary antibody solution from gels.
27. Wash gels 3×10 min with 500 µl PBS containing Tween 20 (0.1%) at room temperature with gentle agitation.
28. Transfer gels into 250 ml lidded amber jars filled with ∼100 ml H_2_O. Incubate for 30 min at room temperature (Fig. 4 step 10; Fig. 5J).
29. Exchange H_2_O and incubate 30 min at RT.
30. Repeat exchange of H_2_O and incubate for 1 h at room temperature (Fig. 4 step 10).
  o Note: gels can be stored in H_2_O at room temperature (protected from light) and imaged up to a week later. Image quality deteriorates quickly, so it is best to image the next day.
31. To estimate the gel expansion factor, remove the gel from H_2_O and place on graph paper (Fig. 5L). Mark paper and measure gel diameter with ruler.
32. Remove excess water from the gel by using a spatula and tilting to allow water to run off.
33. Transfer the gel to a poly-L-lysine-coated glass-bottom imaging dish (Fig. 4 step 11; Fig. 5M).
  o Note: poly-L-lysine-coated dishes should be prepared by spreading 1 ml poly-L-lysine across the glass surface of the dish, letting sit for 30 min, removing excess liquid and allowing to dry.
34. Add 1–2 drops of H_2_O to the top of the gel and cover the imaging chamber to reduce evaporation.
  o Note: if you experience gel drift, one can secure the gel using VALAP as stated in the S2 cell protocol above.
35. Image (Fig. 5M).

### Additional methods

#### Calculating expansion factor

There are two ways to calculate the expansion factor of a given experiment or gel. The first is to use an estimate based on the pre-expanded and post-expanded gel dimensions (Fig. 5L). This can then be used to gain a rough measurement of the true biological distance. For example, if one is imaging a sample using a 100× objective and a CCD camera with a 6.5 μm pixel, then each pixel represents 65 nm of true ‘biological distance’. If the pre-expanded and post-expanded gel ratio indicates a 4× expansion factor, then each pixel on the image would represent 16.25 nm of true biological distance. Using this method of calculating expansion factor, our cultured cell protocol yields an average expansion factor of 4×, whereas expansion of *Drosophila* testes yields an average expansion factor of 4.6×.

The second method of estimating true biological distance in an expanded sample is using a known distance within a cell. We refer researchers to previous work for a thorough expansion factor analysis using the diameter of the spermatid centriole (Wainman, 2021). Briefly, one can divide the measured diameter of a particular centriole protein such as Asl in the expanded image by the actual Asl diameter determined by non-expanded methods. For our spermatid expansion estimates for Figs 1 and 2, we measured the diameter of centrioles stained for Asl (guinea pig antibody against amino acids 630–994) and Ana1 (chicken antibody against amino acids 876–1310). Using the peak-to-peak measurement of line scans across the centriole width, we found that the Asl measured diameter was 1215±167 nm (*n*=13; mean±s.d.) and the Ana1 diameter was 1015±202 nm (*n*=7). Dividing the Asl measurements by the known diameter of the Asl C terminus (∼230 nm; Fu et al., 2016) and the Ana1 measurements by the Ana1 central region (∼200 nm; Fu et al., 2016) yielded expansion factors of 5.3× and 5.1×, respectively. We then used the average of the two expansion factors (5.2×) to indicate the true biological distance using an image scale bar.

Measuring expansion factor does not accurately provide a measurement of image resolution. Expansion resolution can be estimated using the theoretical resolution of the optical setup combined with the expansion factor. Alternatively, one can perform a more direct measurement of resolution using fluorescent bead analysis; however, this is not a practical approach for most studies.

Measuring true biological distances in expanded samples will always present a challenge, and the researcher should take care not to overinterpret small changes in distance. Studies that require extremely accurate measurements of distance should be carefully designed. It is also critically important that one be aware that the formaldehyde:acrylamide ratio in the Anchoring solution influences isotropic expansion. For experiments where accurate cellular measurements are critical for the biological conclusion, one must show that the protocol provides isotropic expansion. Refer to Gambarotto et al. (2019) and Gambarotto et al. (2021) for detailed discussion.

#### Statistical analysis

For comparison between five groups, a one-way ANOVA was used (Fig. 2E). Sample sizes are denoted in the figure legends. All statistics were performed using GraphPad Prism 10.0.3 (GraphPad Software, Boston, MA, USA). Error bars represent the mean±s.d. for each graph.

#### Antibody production

Ana1 amino acids 876–1310 were expressed in BL21(DE3) (Thermo Fisher) with a 6×His N-terminal tag using pDest17 vector (Thermo Fisher). Protein was expressed for 3 h at 37°C and cells frozen at −80°C. Cell pellets were thawed in His-washing buffer (58 mM Na_2_HPO_4_, 17 mM NaH_2_PO_4_, 68 mM NaCl, 20 mM imidazole and 1% Triton X-100) and sonicated for ∼2 min at 40% power (Branson Sonifier 450). Lysate was spun for 13 min at ∼10,000 ***g*** at 4°C. Soluble lysate was incubated on His-Pur Cobalt resin (Thermo Fisher) overnight at 4°C. Resin was washed three times with His-washing buffer and purified protein was eluted in His-elution buffer (58 mM Na_2_HPO_4_, 17 mM NaH_2_PO_4_, 68 mM NaCl, 300 mM imidazole, 1% Triton X-100 and 10% glycerol). Protein was sent to Pocono Rabbit Farm and Laboratory, PA, USA, where antibodies were raised in two chickens.

*Plodia* SYCP3 antibody was generated by first identifying the *Plodia* SYCP3 protein via BLAST search using the *Bombyx mori* SYCP3 protein as bait (Xiang et al., 2023). Full-service antibody production (SYCP3 protein expression, purification and immunization) was performed by Thermo Scientific. Antibodies were raised in guinea pigs.

#### Cell culture, transfection and dsRNA knockdown

*Drosophila* S2 cells (Invitrogen) were cultured in Sf-900II SFM medium (Life Technologies) supplemented with penicillin and streptomycin. dsRNA and DNA plasmid were transfected into S2 cells by nucleofection as previously described (Rogers and Rogers, 2008). The RNAi experiments were performed for 7 days. Oligonucleotides used were: Sas6-RNAi-For: 5ʹ-TAATACGACTCACTATAGGGATGTGGCCTCCAGGGAGC-3ʹ, Sas6-RNAi-Rev: 5ʹ-TAATACGACTCACTATAGGGTGATGTTGGCCACATCCCC-3ʹ, Ana2-RNAi-For: 5ʹ-TAATACGACTCACTATAGGGTGGCGGCTCTGGTATCCC-3ʹ, Ana2-RNAi-Rev: 5ʹ-TAATACGACTCACTATAGGGTCCAGTTGCTCCTCGGG-3ʹ. Briefly, ∼5 × 10^6^ cells were pelleted by centrifugation. Cell pellets were resuspended in 100 µl of transfection solution (5 mM KCl, 15 mM MgCl_2_, 120 mM sodium phosphate, and 50 mM D-mannitol, pH 7.2) containing 40 µg of dsRNA and then transfected again at day 4 with 30 µg of dsRNA for a total of 70 µg (10 µg/day) or 2 µg of Plk4-GFP plasmid DNA, transferred to a cuvette (2 mm gap size), and then electroporated using a Nucleofector 2b (Lonza), program G-030. Transfected cells were diluted immediately with 0.4 ml SF-900 II medium and plated in a 6-well cell culture plate containing 1 ml of fresh medium. Cells were allowed 24 h to recover before additional handling. Expression of the Plk4-GFP construct was induced by the addition of 0.25 mM CuSO_4_ to the culture medium. Plk4-GFP cells were plated 24 h after induction.

#### Immunoblotting

RNAi-treated cells were lysed in lysis buffer (50 mM Tris-HCl pH 7.2, 150 mM NaCl, 0.5% Triton X-100, 1 mM DTT, and 0.1 mM PMSF), and the concentrations were determined by Bradford protein assay (Bio-Rad), followed by the addition of Laemmli sample buffer. Extracts were boiled for 5 min and stored at −20°C. Samples of equal total protein were resolved by SDS-PAGE, transferred onto nitrocellulose (GE Healthcare), probed with primary rabbit anti-Sas6 (Rogers laboratory,1:1000) and rabbit anti-Ana2 (Rogers laboratory, 1:1000) and mouse anti-α-tubulin monoclonal DM1A (Thermo Fisher Scientific, 1:3000) antibodies, and secondary anti-rabbit and anti-mouse IRDye 800CW antibodies (Li-Cor Biosciences) prepared according to the manufacturer's instructions and used at 1:3000 dilution. Antibodies were diluted in western blocking buffer (5% milk in PBS, 0.1% Tween 20). Nitrocellulose membranes were then scanned on a LiCor Odyssey CLx imager (Li-Cor Biosciences).

#### Immunofluorescence microscopy

For SRM imaging experiments, S2 cells were spread on ConA-coated glass-bottom plates and fixed in ice cold methanol at −20°C for 15 min. Cells were washed with PBS containing 0.1% Triton X-100 and blocked in NGS blocking buffer (5% NGS in PBS, 0.1% Triton X-100) for 30 min at room temperature. Primary antibodies were diluted in NGS blocking buffer and slides were incubated overnight at 4°C. Antibodies were used at the following dilutions: rabbit anti-Ana2 (this study), chicken anti-Cep97 (1:1000), rat anti-Asl (1:1000), and rabbit anti-Sas6 (1:500), mouse anti-acetylated tubulin (1:1000, Sigma-Aldrich), human anti-α-tubulin (1:1000, ABCD antibodies), and human anti-β tubulin (1:1000, ABCD antibodies) (see Table S1). Slides were washed 3× with PBS containing 0.1% Triton X-100. Secondary antibodies and 3.2 μM Hoechst 33342 (Thermo Fisher Scientific) were diluted in NGS blocking buffer and slides were incubated for 30 min. Secondary antibodies were used at the following dilutions: donkey anti-host animal AlexaFluor 488 (Thermo Fisher Scientific, 1:1500), anti-host animal Rhodamine Red-X (Jackson Immunoresearch, 1:1500) and anti-host animal AlexaFluor Plus 647 (Thermo Fisher Scientific, 1:1500) (see Table S1). Slides were then washed 3× with PBS containing 0.1% Triton X-100 and mounted with homemade mounting medium (PBS, 90% glycerol and 0.1 M propyl gallate).

#### *Drosophila* testes preparation for conventional microscopy

Testes were dissected in Schneider's medium (Thermo Fisher Scientific) with antibiotic-antimycotic (Thermo Fisher Scientific) and fixed in 9% PFA at room temperature for 15–20 min. Testes were washed in PBS containing 0.3% Triton X-100 (PBST) then blocked for 1–2 h at room temperature in PBST with 5% NGS. Samples were incubated in primary antibody in blocking solution at 4°C overnight (guinea pig anti-Asterless, 1:10,000; chicken anti-Ana1, 1:1000; chicken anti-Yuri, 1:100; rabbit anti-Sas6, 1:500; Table S1). Samples were then washed 3×10 min each in PBST, then incubated in secondary antibody in blocking solution (1:1000 dilution) for 4 h at room temperature. After washing 3×10 min each in PBST, samples were mounted in Aqua-Poly/Mount (Polysciences) for confocal imaging.

#### *Drosophila* ovary preparation for conventional microscopy and ExM

Ovaries from adult mated *w^1118^* females were dissected in room-temperature 1× PBS, and mature egg chambers and eggs were removed (10 ovary pairs per experiment) and placed in cold PBS until all dissections were complete. Ovaries were then fixed in 4% PFA for 15 min at room temperature. Ovaries were then washed 2× in PBS-T (PBS with 0.1% Triton-X 100) for 5 min each. Subsequently, a dual permeabilization was performed: (1) 10 min in 100% methanol on ice followed by two 5 min washes in PBS-T, then (2) PBS with 0.5% Triton-X 100 for 15 min at room temperature. After permeabilization, ovaries were blocked in 2% BSA in PBS-T for 1 h at room temperature. Ovaries were incubated in primary antibodies at 4°C overnight [mouse anti-C(3)G IA8, 1:500; Table S1]. The next day, ovaries were washed thrice in PBS-T for 20 min before incubating in secondary antibodies for 2 h at room temperature (CF680R donkey anti-mouse, 1:500; Biotium). Ovaries were then DAPI stained for 10 min at room temperature, washed in PBS-T for 3×5 min, and mounted in Prolong Diamond (Invitrogen). Slides were left to cure 24–72 h with magnets holding down the coverslip before sealing with nail polish. All washes and incubations were performed with rotation unless otherwise noted.

For ovary expansion, the only change to the ExM tissue protocol was the use of 10 μl MS instead of 15 μl in step 10. Antibodies for ovary ExM were diluted in 2% BSA in PBS blocking solution and used as the following concentrations: mouse anti-C(3)G IA8 (1:250), mouse anti-C(3)G IG5 (1:250), mouse anti-C(3)G 5G4 (1:250), AlexaFluor 488-conjugated secondary antibody (Invitrogen, 1:250).

#### *Plodia* testes preparation for conventional microscopy and ExM

Testes were dissected from wild-type bFog late 4th/early 5th instar *Plodia* larvae in 1× PBS on ice (Heryanto et al., 2022), washed twice in 1× PBS and fixed in 4% PFA for 6 min on a siliconized coverslip. After fixation, testes were manually squashed between siliconized coverslip and a poly-L-lysine-coated glass slide. Slides carrying testes squashes were snap frozen before removing the coverslips and washed in 1× PBS for 5 min, then 0.1% PBST for 5 min twice. Slides were first subjected to permeabilization with 100% methanol on ice for 10 min, washed in 0.1% PBS-T, then permeabilized again in 0.5% PBS-T at room temperature for 15 min before blocking in 5% milk at room temperature for 1 h. Primary antibodies (this study) were incubated on slides under parafilm coverslips overnight at 4°C. Slides were then washed thrice in 0.1% PBS-T before adding secondary antibodies (1:250, goat anti-guinea pig AlexaFluor 488, Invitrogen) for a 1 h room temperature incubation. After washing off secondary antibodies, slides were DAPI stained, washed thrice in 0.1% PBS-T, mounted in Slow-Fade (Invitrogen), and sealed with nail polish before imaging.

For *Plodia* testes expansion, the protocol for *Drosophila* testes was followed precisely. Antibodies for testes ExM were diluted in 2% BSA in PBS blocking solution and used as the following concentrations: guinea pig anti-SYCP3, 1:250; anti-guinea pig AlexaFluor 488 (Invitrogen), 1:250.

#### Microscopy

SRM of S2 cells was performed using a Yokogawa CSU-W1 SoRa spinning disk mounted on a Nikon Ti-2 Eclipse microscope equipped with a sCMOS Kinetix camera (Photometrics) and a Nikon 100× silicone-immersion objective (NA 1.35) with a 2.8× zoom. Microscope control was performed with the Elements software package (Nikon). Expanded samples were imaged using a Nikon 100× silicone-immersion objective (NA 1.35). All images were processed and analyzed using Nikon Elements software (Nikon Instruments).

For *Drosophila* tissue (testes and ovaries), confocal images were primarily acquired using a Nikon Eclipse Ti2 (Nikon Instruments, Melville, NY, USA) with a Yokogawa CSU-W1 spinning-disk confocal head (Yokogawa Life Science, Sugar Land, TX, USA) equipped with a Prime BSI CMOS camera (Teledyne Photometrics, Tucson, AZ, USA) and Nikon Elements software (Nikon Instruments). Expanded samples were imaged using a 100×/1.35 NA silicone immersion objective and non-expanded samples were imaged using a 100×/1.49 NA oil immersion TIRF objective. All images were analyzed and processed in FIJI (ImageJ, National Institutes of Health, Bethesda, MD, USA).

For *Plodia* testes, ExM and non-ExM samples were imaged on a Leica Stellaris 8 laser-scanning confocal/Leica DMi8 widefield hybrid system, equipped with APO 63×/1.40 Oil objective (Leica), K8 CMOS Monochrome Camera, LED8 light source, and with DAPI/FITC/CY3/ CY5/CY7 filter cubes. ExM images were post-processed with Leica Lightning Deconvolution and non-ExM images were post-processed using Leica Thunder Deconvolution software.

#### *Drosophila melanogaster* stocks and husbandry

All strains used are listed in Table S1. Fly stocks were maintained on Bloomington-recipe fly food (LabExpress). Crosses were performed at 25°C. The following fly strains were used in this study: *y,w* (gift from Mark Peifer, University of North Carolina, Chapel Hill, NC, USA), *UAS-Ana1::tdTomato* (gift from Tomer Avidor-Reiss, University of Toledo, Toledo, OH, USA), *Spag4::GFP* (Bloomington *Drosophila* Stock Center), *Ubi-Spag4::TR* (this study; BestGene Inc., Chino Hills, CA, USA) and *Ubi-Cep104::GFP* (Ryniawec et al., 2023). Transgenic flies were generated using standard p-element transformation (Bestgene, Inc.).

#### Cloning

Spag4, as described by Kracklauer et al. (2010), was synthesized (Twist Bioscience) into pTwist ENTR with the following linker sequences used to make it similar to pENTR/D-TOPO vector (Thermo Fisher): 5′ linker CCGCGGCCGCCCCCTTCACC before the ATG of Spag4, 3′ linker AAGGGTGGGCGCGCCG after the last coding amino acid of Spag4. Spag4 was moved to pCaSpeR4 containing an attB recombination site, Ubiquitin promoter, a Gateway cassette and TagRFP (pCaUWTR) by LR Clonase II reaction.

## Supplementary Material

10.1242/joces.264338_sup1 Supplementary information
