# Supplementary material for: Visualizing Drosophila centrioles by expansion microscopy
Source: J Cell Sci. 2026 Jan 19;139(1):jcs264338. doi: 10.1242/jcs.264338 (PMC12863302; doi:10.1242/jcs.264338)
Supplement: Supplementary information [file joces-139-264338-s1.pdf]

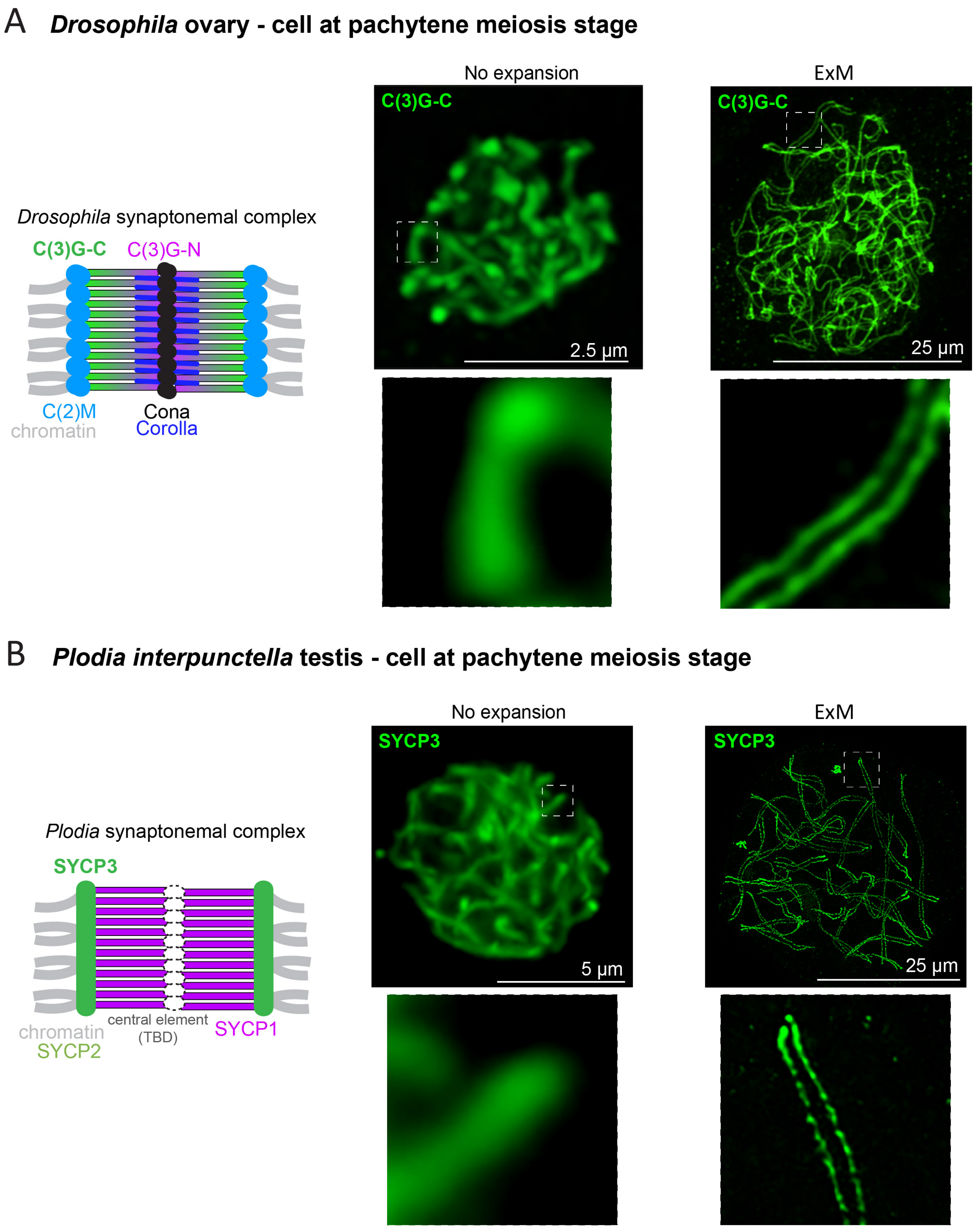

Figure S1

Journal of Cell Science • Supplementary information

Fig. S1. ExM microscopy of synaptonemal complexes in *Drosophila* and *Plodia*

(A) *Drosophila* Ovary. (Left) Schematic of the synaptonemal complex in *Drosophila melanogaster* showing lateral elements (light blue), transverse filaments (green and purple), and central elements (black and dark blue). (Fluorescent micrographs) Pachytene cells from the germarium of whole-mount *Drosophila* ovaries stained for the C-terminus of C(3)G and imaged by confocal microscopy. Left is no expansion, right is U-ExM. Bottom panels are a zoomed images of the boxed regions. Scale bars are as indicated, expansion factor was calculated as 4.5x using the average measured distance of 450nm in ExM between C(3)G parallel lines, and the known distance of 100nm.

(B) *Plodia* testes. (Left) Schematic of the synaptonemal complex in the *Plodia interpunctella*, showing lateral elements (green), transverse filaments (purple), and the predicted location of central elements (empty dashed circles; no proteins identified to date). (Fluorescent micrographs) Cell at the pachytene stage of meiosis from *Plodia* whole testes squash preps stained for SYCP3 and imaged by widefield microscopy for the non-expanded sample, and confocal microscopy for the expanded sample. Bottom panels are a zoomed images of the boxed regions. Scale bars are as indicated, expansion factor was calculated as 4x using ratio of pre and post-expanded gels.

Journal of Cell Science • Supplementary information

Table S1.

REAGENT or RESOURCE	SOURCE	IDENTIFIER
**Antibodies**		
Rat polyclonal anti-Asl antibody	Rogers Lab	(Boese et al., 2018)
Rabbit polyclonal anti-Plp antibody	Rogers Lab	(Rogers et al., 2008)
Chicken polyclonal anti-Cep97 antibody	Rogers Lab	(Ryniawec et al., 2023)
Rat polyclonal anti-Cep135 antibody	Rogers Lab	(McLamarrah et al., 2018)
Rabbit polyclonal anti-Sas6 antibody	Rogers Lab	(Rogers et al., 2008)
Rabbit polyclonal anti-Ana2 antibody	Rogers Lab	This study
Human monoclonal anti-α-tubulin antibody	ABCD antibodies	ABCD_AA345
Human monoclonal anti-β-tubulin antibody	ABCD antibodies	ABCD_AA344
Goat polyclonal anti-GFP antibody	Rockland	Cat#600-101-215
Mouse monoclonal anti-acetylated tubulin	Sigma Aldrich	Cat#T6793
Guinea Pig anti-Asl antibody	Rusan Lab	(Klebba et al., 2013)
Chicken anti-Ana1 antibody	This study	N/A
Chicken anti-Yuri 4012 antibody	Beckingham Lab	(Texada et al., 2008)
Rabbit polyclonal anti-GFP antibody IgG	AbCam	Cat#AB290
Rabbit polyclonal anti-RFP antibody IgG	Rockland	Cat#600-401-379
Mouse anti-C(3)G IA8	Hawley Lab	(Anderson et al., 2005)
Mouse anti-C(3)G IG5	Hawley Lab	(Anderson et al., 2005)
Mouse anti-C(3)G 5G4	Hawley Lab	(Anderson et al., 2005)
Guinea pig anti-SYCP3 antibody	This study	N/A

Journal of Cell Science • Supplementary information

AlexaFluor 488 donkey anti-rabbit IgG	Thermo Fisher Scientific	Cat#A21206
Alexa Fluor 488 AffiniPure donkey anti-goat IgG	Jackson Immunoresearch	Cat#705-545-003
Alexa Fluor 488 goat anti-chicken IgG	Thermo Fisher Scientific	Cat#A11039
Alexa Fluor 488 goat anti-chicken IgY	Thermo Fisher Scientific	Cat#32931
Alexa Fluor 488 goat anti-rabbit IgG	Invitrogen	Cat#A11008
Alexa Fluor 488 donkey anti-mouse IgG	Invitrogen	Cat#A21202
Alexa Fluor 488 goat anti-guinea pig IgG	Invitrogen	Cat#A11073
Rhodamine Red-X AffiniPure goat anti-rat IgG	Jackson Immunoresearch	Cat#112-295-143
Rhodamine Red-X AffiniPure goat anti-mouse IgG	Jackson Immunoresearch	Cat#115-295-003
Alexa Fluor 568 goat anti-guinea pig IgG	Thermo Fisher Scientific	Cat#A11075
Alexa Fluor Plus 647 goat anti-chicken IgG	Thermo Fisher Scientific	Cat#A32933
Alexa Fluor 647 goat anti-guinea pig IgG	Thermo Fisher Scientific	Cat#21450
ATTO 647 goat anti-rabbit IgG	Sigma Aldrich	Cat#40839
CF 680R donkey Anti-Mouse IgG	Biotium	Cat#20194-1
**Bacterial Strains**		
GC10 Competent Bacteria	Genesee Scientific	Cat#42–659 or Cat# 42–661
**Chemicals, peptides, and recombinant proteins**		
Normal Goat Serum	Sigma Aldrich	Cat#G9023
Triton X-100	Thermo Fisher Scientific	Cat#BP151-500

Journal of Cell Science • Supplementary information

DAPI	Thermo Fisher Scientific	Cat#D1306 or Cat#D21490
Hoechst 33342	Life Technologies	Cat#H3570
Sf-900 II SFM	Gibco	Cat#10902-088
Penicillin Streptomycin Solution	Corning	Cat#30-002-CI
Sodium Acrylate 97%	Pfaultz & Bauer	Cat#S03880
Ammonium Persulfate	Fisher	Cat#BP179-25
TEMED	Sigma	Cat#T9281
Poly-ʟ-Lysine Hydrobromide	Sigma	Cat#A-005-C
Acrylamide	Sigma	Cat#A4058-100mL
Bis-Acrylamide	Sigma	Cat#M1533-25mL
Formaldehyde	Sigma	Cat#F8775-25mL
Poly-L-Lysine	Sigma	Cat#P4707-50mL
Schneider's *Drosophila* Media	Thermo Fisher Scientific	Cat#21720-024
Antibiotic-Antimycotic	Thermo Fisher Scientific	Cat#15240-062
Slow Fade^{TM} Diamond Antifade Mountant	Invitrogen	Cat#S36972
**Critical Commercial Assays**		
Gateway LR II Clonase	Thermo Fisher Scientific	Cat#11791100
**Deposited data**		
DOI - 10.25444/nhlbi.30286972		
**Experimental models: Cell lines**		
D. melanogaster: Cell line S2	Thermo Fisher Scientific	
**Experimental models: Organisms/strains**		

D. melanogaster: y,w	Gift from Mark Peifer, UNC Chapel Hill	N/A
D. melanogaster: UAS-Ana1::tdTomato	Avidor-Reiss Lab	(Blachon et al., 2008)
D. melanogaster: Spag4::GFP	Bloomington Drosophila Stock Center	Cat#29975
D. melanogaster: Ubi-Spag4::TR	This study	N/A
D. melanogaster: Ubi-Cep104::GFP	Rusan Lab	(Ryniawec et al., 2023)
**Recombinant DNA**		
pMT/V5 His C Plk4-GFP	Rogers Lab	Rogers Lab
pTwist ENTR-Spag4	This study	N/A
pCaUWTR	This study	N/A
pCaUWTR-Spag4	This study	N/A
**Software and algorithms**		
Prism 7	GraphPad	www.graphpad.com/scientificsoftware/prism/
Elements	Nikon	https://www.microscope.healthcare.nikon.com/products/software/nis-elements/viewer
FIJI / ImageJ	NIH	http://fiji.sc/
Adobe Illustrator	Adobe	https://www.adobe.com/in/products/illustrator.html
Adobe Photoshop	Adobe	https://www.adobe.com/products/photoshop.html
**Other**		
4 mm biopsy puncher	Integra	Cat#33-34

Journal of Cell Science • Supplementary information

4 mm biopsy puncher	Ted Pella	Cat#15110-40
25mm round coverslips	Nest	Cat#801009
12 mm round coverslips	Fisher	Cat#12-545-81
FluoroDish	World Precision Instrument	Cat#FD35-100
Dumont #5 Forceps	Fine Science Tools	Cat#11252-20

Supplemental references

Anderson, L.K., S.M. Royer, S.L. Page, K.S. McKim, A. Lai, M.A. Lilly, and R.S. Hawley. 2005. Juxtaposition of C(2)M and the transverse filament protein C(3)G within the central region of Drosophila synaptonemal complex. *Proc Natl Acad Sci U S A*. 102:4482–4487.

Blachon, S., J. Gopalakrishnan, Y. Omori, A. Polyanovsky, A. Church, D. Nicastro, J. Malicki, and T. Avidor-Reiss. 2008. Drosophila asterless and vertebrate Cep152 Are orthologs essential for centriole duplication. *Genetics*. 180:2081–2094.

Boese, C.J., J. Nye, D.W. Buster, T.A. McLamarrah, A.E. Byrnes, K.C. Slep, N.M. Rusan, and G.C. Rogers. 2018. Asterless is a Polo-like kinase 4 substrate that both activates and inhibits kinase activity depending on its phosphorylation state. *Mol Biol Cell*. 29:2874–2886.

Klebba, J.E., D.W. Buster, A.L. Nguyen, S. Swatkoski, M. Gucek, N.M. Rusan, and G.C. Rogers. 2013. Polo-like kinase 4 autodestructs by generating its Slimb-binding phosphodegron. *Curr Biol*. 23:2255–2261.

McLamarrah, T.A., D.W. Buster, B.J. Galletta, C.J. Boese, J.M. Ryniawec, N.A. Hollingsworth, A.E. Byrnes, C.W. Brownlee, K.C. Slep, N.M. Rusan, and G.C. Rogers. 2018. An ordered pattern of Ana2 phosphorylation by Plk4 is required for centriole assembly. *J Cell Biol*. 217:1217–1231.

Rogers, G.C., N.M. Rusan, M. Peifer, and S.L. Rogers. 2008. A multicomponent assembly pathway contributes to the formation of acentrosomal microtubule arrays in interphase Drosophila cells. *Mol Biol Cell*. 19:3163–3178.

Ryniawec, J.M., M.R. Hannaford, M.E. Zibrat, C.J. Fagerstrom, B.J. Galletta, S.E. Aguirre, B.A. Guice, S.M. Dean, N.M. Rusan, and G.C. Rogers. 2023. Cep104 is a component of the centriole distal tip complex that regulates centriole growth and contributes to Drosophila spermiogenesis. *Curr Biol*. 33:4202–4216 e4209.

Texada, M.J., R.A. Simonette, C.B. Johnson, W.J. Deery, and K.M. Beckingham. 2008. Yuri gagarin is required for actin, tubulin and basal body functions in Drosophila spermatogenesis. *J Cell Sci*. 121:1926–1936.

Journal of Cell Science • Supplementary information
